# Discovery and characterization of a highly efficient enantioselective mandelonitrile hydrolase from *Burkholderia cenocepacia* J2315 by phylogeny-based enzymatic substrate specificity prediction

**DOI:** 10.1186/1472-6750-13-14

**Published:** 2013-02-18

**Authors:** Hualei Wang, Huihui Sun, Dongzhi Wei

**Affiliations:** 1 State Key Laboratory of Bioreactor Engineering, New World Institute of Biotechnology, East China University of Science and Technology, Shanghai 200237, People’s Republic of China

**Keywords:** (R)-(−)-mandelic acid, Nitrilase, *Burkholderia cenocepacia* J2315, Substrate specificity prediction, Enantioselective hydrolysis

## Abstract

**Background:**

A nitrilase-mediated pathway has significant advantages in the production of optically pure (R)-(−)-mandelic acid. However, unwanted byproduct, low enantioselectivity, and specific activity reduce its value in practical applications. An ideal nitrilase that can efficiently hydrolyze mandelonitrile to optically pure (R)-(−)-mandelic acid without the unwanted byproduct is needed.

**Results:**

A novel nitrilase (BCJ2315) was discovered from *Burkholderia cenocepacia* J2315 through phylogeny-based enzymatic substrate specificity prediction (PESSP). This nitrilase is a mandelonitrile hydrolase that could efficiently hydrolyze mandelonitrile to (R)-(−)-mandelic acid, with a high enantiomeric excess of 98.4%. No byproduct was observed in this hydrolysis process. BCJ2315 showed the highest identity of 71% compared with other nitrilases in the amino acid sequence. BCJ2315 possessed the highest activity toward mandelonitrile and took mandelonitrile as the optimal substrate based on the analysis of substrate specificity. The kinetic parameters *V*_max_, *K*_m_, *K*_cat_, and *K*_cat_/*K*_m_ toward mandelonitrile were 45.4 μmol/min/mg, 0.14 mM, 15.4 s^-1^, and 1.1×10^5^ M^-1^s^-1^, respectively. The recombinant *Escherichia coli* M15/BCJ2315 had a strong substrate tolerance and could completely hydrolyze mandelonitrile (100 mM) with fewer amounts of wet cells (10 mg/ml) within 1 h.

**Conclusions:**

PESSP is an efficient method for discovering an ideal mandelonitrile hydrolase. BCJ2315 has high affinity and catalytic efficiency toward mandelonitrile. This nitrilase has great advantages in the production of optically pure (R)-(−)-mandelic acid because of its high activity and enantioselectivity, strong substrate tolerance, and having no unwanted byproduct. Thus, BCJ2315 has great potential in the practical production of optically pure (R)-(−)-mandelic acid in the industry.

## Background

Optically pure 2-hydroxycarboxylic acids are important intermediates in the pharmaceutical and fine chemical industries [1-4]. (R)-(−)-mandelic acid is one of the important 2-hydroxycarboxylic acids, which is widely used for the production of semisynthetic cephalosporins [5], penicillins [6], antitumor agents [7], and antiobesity agents [8]. It is also used as a universal acidic chiral resolving agent for the resolution of racemic alcohols and amines [9].

Several methods have been proposed for the production of optically pure (R)-(−)-mandelic acid [4,10]. Among these methods, nitrilase-mediated pathway is increasingly popular because of its lack of cofactor involvement, cheap starting material in the form of mandelonitrile, high enantioselectivity, and theoretically 100% of the product [10-15]. However, these reported nitrilases either have low enantioselectivity or low specific activity toward mandelonitrile [16]. Furthermore, some also produce a byproduct in the form of mandelamide [16,17]. Therefore, an ideal nitrilase that can efficiently hydrolyze mandelonitrile to optically pure (R)-(−)-mandelic acid without the unwanted byproduct is needed.

Several approaches have been developed to discover novel nitrilases toward mandelonitrile [18-22]. Among these approaches, an enrichment culture [19] and the metagenome approach [20] have been used successfully. However, these methods require screening a large number of clones, and are thereby time consuming. Considering that the number of genes increases exponentially based on an automated genome annotation in the database, genome mining has become increasingly popular in the recent years. Researchers can easily find many genes with a defined function, such as nitrilase, from databases, such as GenBank, Pfam, and Brenda. Nitrilases of interest can be discovered more efficiently by combining the existing methods with substrate specificity prediction. Zhu et al. [21] discovered a mandelonitrile hydrolase (nitrilase) by combining traditional mining with the functional analysis of the flanking genes around this nitrilase. This nitrilase was organized in a mandelonitrile metabolic pathway and displayed high activity toward mandelonitrile. Seffernick et al. [22] also discovered a nitrilase and another mandelonitrile hydrolase from *Burkholderia xenovorans* LB400 using computational methods. However, these two nitrilases exhibited no or only slight enantioselectivity in producing (R)-(−)-mandelic acid.

In our study, phylogeny-based enzymatic substrate specificity prediction (PESSP) was introduced for the efficient discovery of an ideal nitrilase to solve the problems of unwanted byproduct production, low enantioselectivity, and specific activity. A novel nitrilase (BCJ2315) was discovered from *Burkholderia cenocepacia* J2315. BCJ2315 could efficiently hydrolyze mandelonitrile to (R)-(−)-mandelic acid with high enantioselectivity. No byproduct was observed in the hydrolysis process. BCJ2315 was cloned and overexpressed in *Escherichia coli* M15, and its catalytic properties were investigated by analyzing its substrate specificity and kinetic parameters. The catalytic efficiency of the recombinant *E. coli* M15/BCJ2315 was also tested in the hydrolyzing mandelonitrile biotransformation to (R)-(−)-mandelic acid to investigate the potential of BCJ2315 further.

## Results and discussion

### Discovery of a predicted mandelonitrile hydrolase subgroup through PESSP

**Figure 1:**
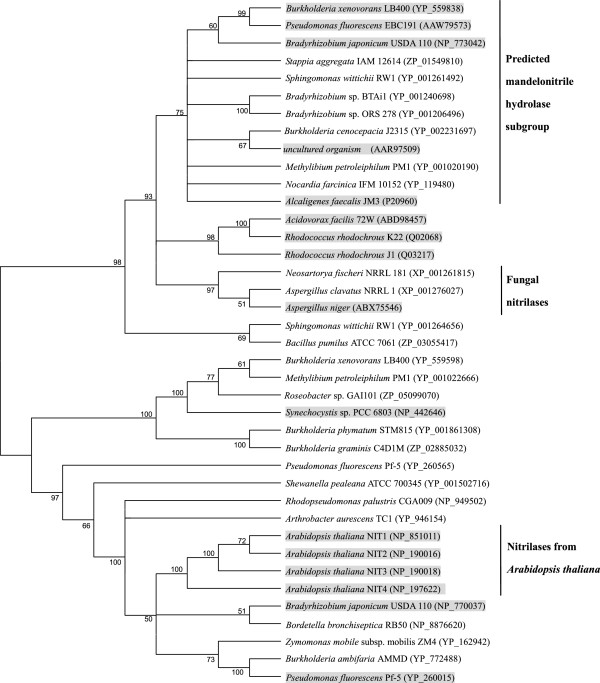
**Unrooted neighbor-joining tree based on the amino acid sequences of the organisms from Table **1**(accession numbers are in parentheses).** A consensus tree was constructed using a bootstrap test with 1000 replications. Bootstrap values greater than 50% are shown at the branch points. The organisms harboring defined nitrilase activity are shadowed.

Based on the screening criteria mentioned in Database mining and sequence analysis section, a total of 39 proteins were chosen for the mandelonitrile hydrolase activity assay (Table 1). These proteins were annotated as nitrilase, putative nitrilase, aliphatic nitrilase, and unnamed protein products. Among the 39 proteins, 16 were experimentally determined to have a nitrilase activity with different substrate specificities. For example, the nitrilase from *Rhodococcus rhodochrous* J1 [23] was designated as an aromatic nitrilase. The nitrilases from *Synechocystis* sp. PCC6803 [24] and *Acidovorax facilis* 72W [25] were specific to aliphatic (di)nitrile. The nitrilase from *Pseudomonas fluorescens* Pf-5 [26] had a regioselective activity toward aliphatic dinitrile. The nitrilases from *Alcaligenes faecalis* JM3 [13], *Pseudomonas fluorescens* EBC191 [17], *Bradyrhizobium japonicum* USDA110 [21], *Burkholderia xenovorans* LB400 [22], and an uncultured organism (nitrilase I, 2A6) [20] were characterized as mandelonitrile hydrolases. Finally, a cluster containing all the defined mandelonitrile hydrolases was found based on the phylogenetic analysis (Figure 1). Seven proteins were not characterized experimentally within this cluster, and their functions remained unclear. Based on the substrate specificities of the defined nitrilases, this cluster was designated as the predicted mandelonitrile hydrolase subgroup. The uncharacterized seven proteins in this subgroup were further studied.

**Table 1:** **Organisms and accession numbers of the putative nitrilases mined from the GenBank database**[27-31]

**Organism**	**GenBank Accession no.**	**Predicted function**	**Defined function**^**b**^	**Identity**^**a**^**(%)**	**Reference**
*Bradyrhizobium japonicum* USDA 110	NP_773042		nitrilase	100	[21]
*Burkholderia xenovorans* LB400	YP_559838		nitrilase	60	[22]
*Pseudomonas fluorescens* EBC191	AAW79573		nitrilase	60	[17]
*Sphingomonas wittichii* RW1	YP_001261492	unnamed protein product		58	This study
*Bradyrhizobium* sp. ORS 278	YP_001206496	aliphatic nitrilase		58	This study
*Bradyrhizobium* sp. BTAi1	YP_001240698	aliphatic nitrilase		57	This study
*uncultured organism*	AAR97509		nitrilase	56	[20]
*Burkholderia cenocepacia* J2315	YP_002231697	putative nitrilase		56	This study
*Methylibium petroleiphilum* PM1	YP_001020190	aliphatic nitrilase		55	This study
*Nocardia farcinica* IFM 10152	YP_119480	nitrilase		55	This study
*Stappia aggregata* IAM 12614	ZP_01549810	nitrilase		53	This study
*Rhodococcus rhodochrous* K22	Q02068		nitrilase	51	[27]
*Rhodococcus rhodochrous* J1	Q03217		nitrilase	50	[23]
*Acidovorax facilis* 72W	ABD98457		nitrilase	49	[25]
*Alcaligenes faecalis* JM3	P20960		nitrilase	47	[13]
*Sphingomonas wittichii* RW1	YP_001264656	unnamed protein product		46	This study
*Neosartorya fischeri* NRRL 181	XP_001261815	nitrilase, putative		45	This study
*Aspergillus clavatus* NRRL 1	XP_001276027	nitrilase, putative		41	This study
*Aspergillus niger*	ABX75546		nitrilase	38	[28]
*Bordetella bronchiseptica* RB50	NP_887662	unnamed protein product		37	This study
*Arthrobacter aurescens* TC1	YP_946154	nitrilase		36	This study
*Bacillus pumilus* ATCC 7061	ZP_03055417	nitrilase		36	This study
*Rhodopseudomonas palustris* CGA009	NP_949502	nitrilase		36	This study
*Pseudomonas fluorescens* Pf-5	YP_260565	carbon-nitrogen family hydrolase		36	This study
*Arabidopsis thaliana*	NP_190018		nitrilase	35	[29]
*Burkholderia graminis* C4D1M	ZP_02885032	nitrilase		35	This study
*Bradyrhizobium japonicum* USDA 110	NP_770037		nitrilase	35	[30]
*Arabidopsis thaliana*	NP_197622		nitrilase	35	[31]
*Methylibium petroleiphilum* PM1	YP_001022666	nitrilase		34	This study
*Synechocystis* sp. PCC 6803	NP_442646		nitrilase	34	[24]
*Burkholderia phymatum* STM815	YP_001861308	unnamed protein product		34	This study
*Arabidopsis thaliana*	NP_190016		nitrilase	34	[29]
*Arabidopsis thaliana*	NP_851011		nitrilase	34	[29]
*Shewanella pealeana* ATCC 700345	YP_001502716	nitrilase		33	This study
*Burkholderia xenovorans* LB400	YP_559598	nitrilase		32	This study
*Roseobacter sp*. GAI101	ZP_05099070	aliphatic nitrilase		32	This study
*Burkholderia ambifaria* AMMD	YP_772488	nitrilase/cyanide hydratase hydratase and apolipoprotein N-acyltransferase		31	This study
*Pseudomonas fluorescens* Pf-5	YP_260015		nitrilase	31	[26]
*Zymomonas mobilis* ZM4	YP_162942	unnamed protein product		30	This study

^a^ BLASTP was performed by comparing the amino acid sequence with that of the mandelonitrile hydrolase (bll6042, NP_773042) from *Bradyrhizobium japonicum* USDA 110.

^b^ The enzymes were experimentally characterized to harbor nitrilase activity.

### Identification of BCJ2315 from the predicted mandelonitrile hydrolase subgroup

The respective genes of the seven proteins were cloned and overexpressed in *E. coli* to verify whether these uncharacterized proteins in the predicted mandelonitrile hydrolase subgroup have a mandelonitrile hydrolase activity. The resulting recombinant histidine (His)-tagged proteins were all soluble and purified to homogeneity for the catalytic activity assay. All the seven enzymes were active toward mandelonitrile and have relatively high enantioselectivity (Table 2). Among these enzymes, BCJ2315 from *Burkholderia cenocepacia* J2315 exhibited the highest specific activity (27.79 U/mg) and enantioselectivity (98.4%). Only the SA12614 produced amide as a reaction byproduct. The sequence analysis of BCJ2315 showed 52%, 59%, 56%, 60%, and 66% identities with the nitrilases from *Alcaligenes faecalis* JM3 [13], *Pseudomonas fluorescens* EBC191 [17], *Bradyrhizobium japonicum* USDA110 [21], *Burkholderia xenovorans* LB400 [22], and an uncultured organism (nitrilase I, 2A6) [20], respectively. The highest identity was 71% compared with nitrilase (2A12) from an uncultured organism discovered by Robertson et al. [20]. To the best of our knowledge, the current study is the first report of nitrilase BCJ2315. BCJ2315 was chosen for further study because it had the highest activity and enantioselectivity toward mandelonitrile.

**Table 2:** Specific activity and enantioselectivity of the nitrilases in predicted mandelonitrile hydrolase subgroup

**Nitrilase**	**Organism**	**Specific activity (U/mg)**	**ee value of acid (%)**	**Amide formation (% of total products)**
BCJ2315	*Burkholderia cenocepacia* J2315	27.79±1	98.4	N.D.
SWRW1	*Sphingomonas wittichii* RW1	4.60±0.3	96.4	N.D.
MPPM1	*Methylibium petroleiphilum* PM1	7.67±0.5	93.4	N.D.
SA12614	*Stappia aggregata* IAM 12614	3.87±0.3	92.8	8
NF10152	*Nocardia farcinica* IFM 10152	10.11±0.7	91.5	N.D.
BSBTAi1	*Bradyrhizobium* sp. BTAi1	5.82±0.5	90.9	N.D.
BS278	*Bradyrhizobium* sp. ORS 278	1.33±0.06	89.7	N.D.

N.D. Not detected.

The rest of the uncharacterized genes outside the predicted mandelonitrile hydrolase subgroup in the phylogenetic tree were also cloned and overexpressed in *E. coli* (Additional file 1: Table S1) so that any other nitrilases with good characteristics toward mandelonitrile would not be missed. After optimizing the expression conditions (induction temperature, Isopropyl-β-D-thiogalactopyranoside (IPTG) concentration, and expression vector/host), all the enzymes were expressed in a soluble form and showed activity toward at least one of the four assayed nitrile substrates (benzonitrile, phenylacetonitrile, acrylonitrile, and succinonitrile). The recombinant His-tagged proteins were purified for the mandelonitrile hydrolase activity assay. Little to no activity was observed in the high-performance liquid chromatography (HPLC) analysis after 12 h of hydrolysis (Additional file 1: Table S1). This result further proved the accuracy of the prediction based on phylogenetic analysis.

### Properties of the purified BCJ2315

**Figure 2:**
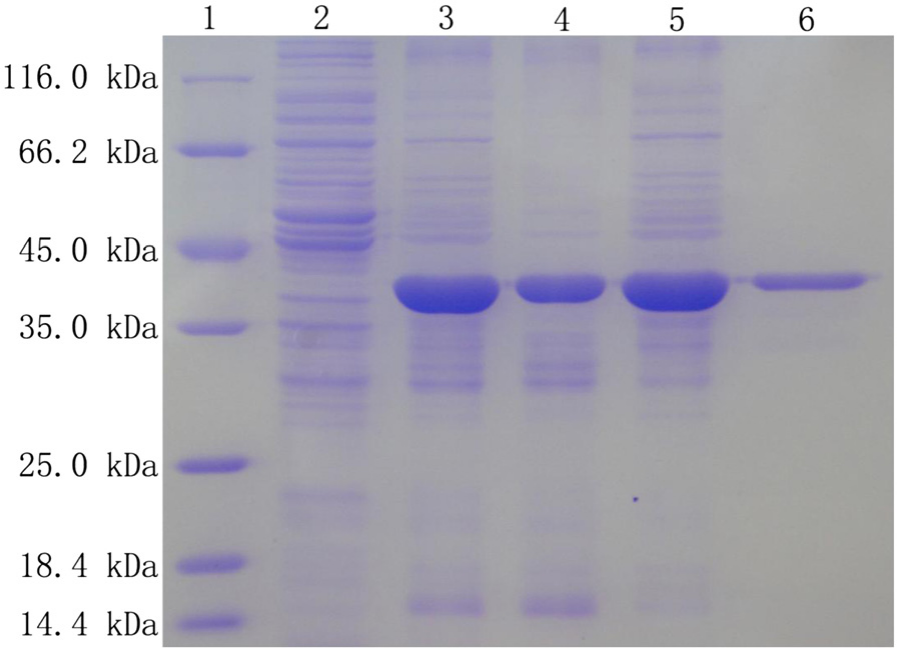
**SDS-PAGE analysis of the nitrilase BCJ2315.** Lane 1: protein marker; Lane 2; whole cell lysates of *E. coli* M15/pQE30; Lane 3: whole cell lysates of *E. coli* M15/BCJ2315; Lane 4: insoluble fractions of the whole cell lysates of *E. coli* M15/BCJ2315; Lane 5: soluble fractions of the whole cell lysates of *E. coli* M15/BCJ2315; Lane 6: purified BCJ2315.

The molecular weight of the purified native BCJ2315 estimated by conducting a gel filtration chromatography was about 450 kDa. BCJ2315 showed one single band on the SDS-PAGE with a molecular weight of 37 kDa (Figure 2). This result indicated that the native BCJ2315 consisted of 12 subunits with identical sizes, which is in agreement with most nitrilases reported with 6 to 26 identical subunits that self-aggregated to form active enzymes [32].

**Figure 3:**
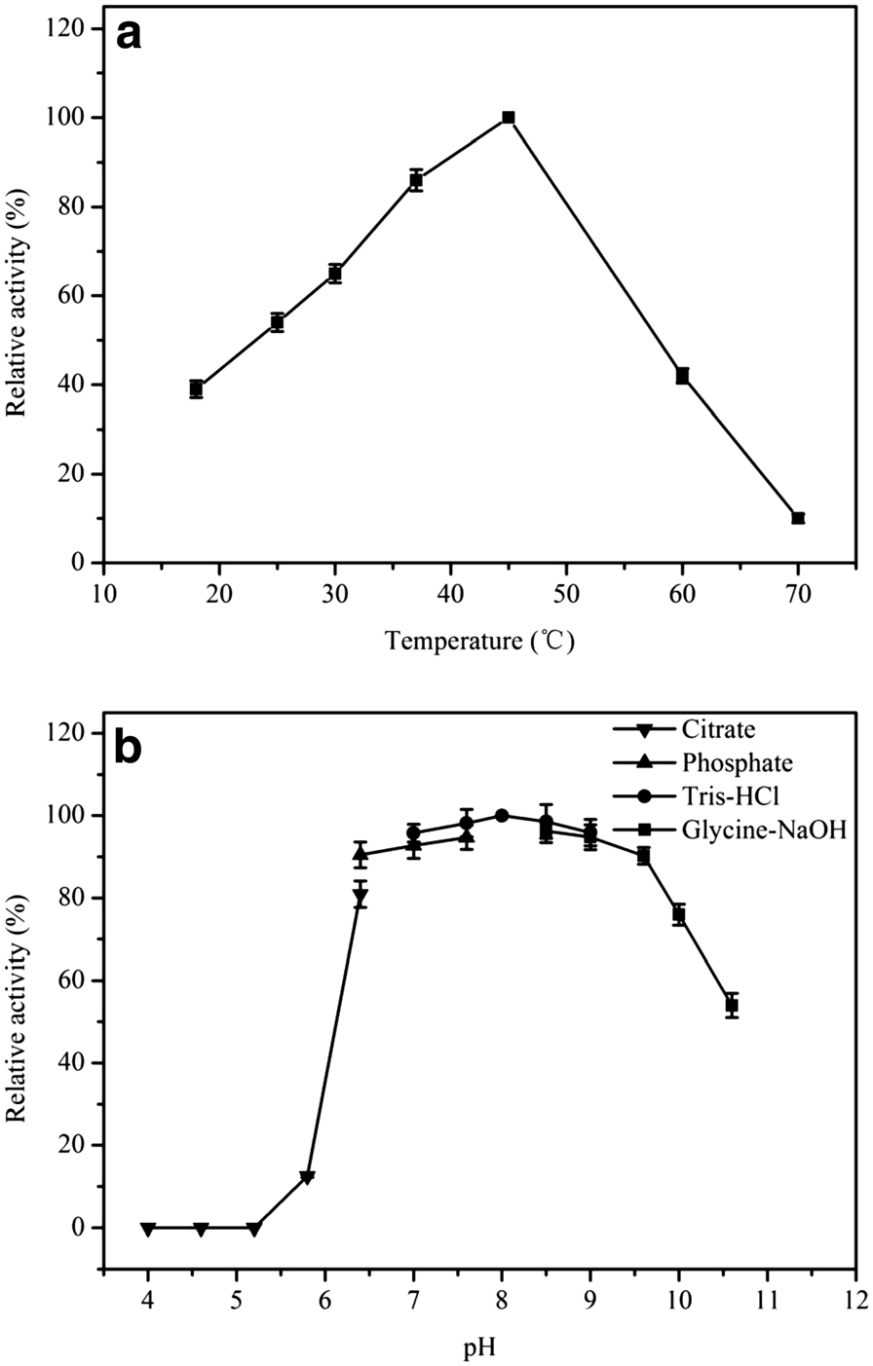
**Effect of temperature and pH on the activity of purified BCJ2315 toward mandelonitrile. (a)** Optimum temperature: enzyme activity was measured at various temperatures (18°C to 70°C) in 100 mM of sodium phosphate buffer (pH 7.0). **(b)** Optimum pH: enzyme activity was measured in different buffers (pH 4.0 to 10.6) at 30°C. The relative activity was expressed as a percentage of the maximum activity under the experimental condition used.

The optimum temperature and pH of the purified BCJ2315 were determined. The optimum temperature was 45°C, as shown in Figure 3a. When the temperature was above 45°C, the activity of BCJ2315 decreased sharply. This behavior is similar to the nitrilases reported from mesophilic organisms, having optimum temperatures ranging from 30°C to 50°C [1,11,12,17,33]. BCJ2315 showed the highest activity at pH 8.0 (Figure 3b). Only small changes in activity were observed between pH 6.4 and 9.6. These variations suggested that BCJ2315 had a relatively broad optimum pH in contrast to other arylacetonitrilases that have a rather narrow optimum pH at neutral or slightly alkaline pH values [11].

The catalytic activity of BCJ2315 toward 24 different nitriles with structural diversity was investigated. Table 3 lists the relative activities determined by quantifying the amount of ammonia released during the hydrolysis. A clear preference of BCJ2315 for arylacetonitriles as substrates indicated that this enzyme is an arylacetonitrilase. A lower activity was also observed with the aliphatic and heterocyclic nitriles. No detectable activities were observed with the aromatic nitriles. BCJ2315 showed the highest activity toward mandelonitrile (8.8 times more than that of phenylacetonitrile), indicating that BCJ2315 is a highly active mandelonitrile hydrolase. Moreover, the activities of other arylacetonitrilases, such as *Alcaligenes faecalis* ATCC 8750 [10], *Alcaligenes* sp. ECU0401 [11], *Alcaligenes faecalis* ZJUTB10 [12], *Alcaligenes faecalis* JM3 [13], and *Pseudomonas putida*[33], toward mandelonitrile are only 12% to 50% of that for phenylacetonitrile.

**Table 3:** Substrate specificity of the purified BCJ2315

**Entry**	**Substrate**	**Relative activity**^**a**^**(%)**
1	Iminodiacetonitrile	21±1
2	acrylonitrile	17±1
3	2-Methylglutaronitrile	25±3
4	Succinonitrile	5±1
5	Fumaronitrile	78±3
6	Malononitrile	17±1
7	Valeronitrile	4±1
8	3-Hydroxyglutaronitrile	N.D.
9	4-Chlorobutyronitrile	11±1
10	Glycolonitrile	69±4
11	3-Phenylpropionitrile	N.D.
12	Benzonitrile	N.D.
13	Cinnamonitrile	N.D.
14	Phenylacetonitrile	100±4
15	2-Chloromandelonitrile	298±7
16	1,2-Phenylenediacetonitrile	37±5
17	2-Phenylbutyronitrile	N.D.
18	alpha-Methylphenylacetonitrile	N.D.
19	mandelonitrile	884±7
20	3-Cyanopyridine	N.D.
21	Thiophene-3-carbonitrile	1±1
22	Indole-3-acetonitrile	20±1
23	2-Cyanopyridine	7±1
24	4-Cyanopyridine	7±1

^a^ Nitrilase activity against various nitriles was measured under standard assay conditions. The activity toward phenylacetonitrile was defined as 100%.

N.D. not detected.

The kinetic parameters of BCJ2315 were determined using mandelonitrile as substrate. The obtained *K*_m_ and *V*_max_ were 0.14 mM and 45.4 μmol/min/mg, respectively. The low value of *K*_m_ indicated that BCJ2315 had high affinity toward mandelonitrile. The *K*_m_-values of other highly enantioselective mandelonitrile hydrolases were one to three orders of magnitude higher (above 3.4 mM) than that of BCJ2315 (Table 4) [11,12,16,33]. The *K*_cat_ and *K*_cat_/*K*_m_ were 15.4 s^-1^ and 1.1×10^5^ M^-1^s^-1^, respectively. BCJ2315 also had a high catalytic efficiency, comparable with that of the nitrilase (bll6402) from *Bradyrhizobium japonicum* USDA110 (1.04×10^5^ M^-1^s^-1^) [21].

**Table 4:** **Kinetic parameters, specific activity, enantioselectivity, and specificity of BCJ2315 compared with other mandelonitrile hydrolases**[33-35]

**Organism**	**Kinetic parameters**		**Specific activity (U/mg)**	**ee value of acid (%)**	**Amide formation (% of total products)**	**Reference**
	***K***_**m**_**(mM)**	***V***_**max**_**(μmol/min/mg)**				
*uncultured organism*^a^	N.A.	N.A.	50	98	N.D.	[34]
*Pseudomonas fluorescens* EBC191	N.A.	N.A.	32.8	31	19	[19]
*Burkholderia cenocepacia* J2315	0.14	45.4	27.79	98.4	N.D.	This study
*Bradyrhizobium japonicum* USDA110^b^	0.26	44.7	24.38	-	N.D.	[21]
*Alcaligenes* sp. ECU0401	21.8	27.9	19	97	N.D.	[11]
*Alcaligenes faecalis* JM3	N.A.	N.A.	12.4	99	N.D.	[13]
*Aspergillus niger* CBS 513.88	11.4	12.4	7.7	99	N.D.	[16], [35]
*Neurospora crassa* OR74A	3.4	9.9	6.1	99	15	[16], [35]
*Pseudomonas putida*	13.39	16.5	3.26	99.9	N.D.	[33]
*Alcaligenes faecalis* ATCC8750	N.A.	N.A.	3.1	100	N.D.	[10]
*Burkholderia xenovorans* LB400	0.084	N.A.	1.523	-	N.D.	[22]
*Alcaligenes faecalis* ZJUTB10	4.74	15.85	N.A.	99	N.D.	[12]

N.A. data not available.

N.D. not detected.

- no enantioselectivity was observed.

^a^ nitrilase I (2A6) was referred here.

^b^ nitrilase (bll6402) was referred here.

BCJ2315 exhibited a relatively high specific activity (27.79 U/mg) among the reported mandelonitrile hydrolases. It has the 2^nd^ highest activity of all known highly enantioselective nitrilases in literature (Table 4). The highest specific activity toward mandelonitrile was observed with nitrilase I (50 U/mg) from an uncultured organism discovered by Robertson et al. Nitrilase I also has an excellent enantioselectivity toward mandelonitrile (enantiomeric excess (ee), 98%) and mandelonitrile derivatives [20,34]. Strangely, these two nitrilases only shared a 66% identity in the amino acid sequence, probably because of the method we used (PESSP). The PESSP method discovered new enzymes based on their substrate specificity toward mandelonitrile, other than the sequence identity. Therefore, despite the low identity between these two nitrilases, they were successfully clustered together into the predicted mandelonitrile hydrolase subgroup. PESSP exhibited an advantage in searching for enzymes with similar characteristics, although these enzymes may be quite different in the amino acid sequence. A higher specific activity was also observed with the nitrilases from *Pseudomonas fluorescens* EBC191 (32.8 U/mg) and *Bradyrhizobium japonicum* USDA110 (24.38 U/mg). However, these two nitrilases had lower ees, and the nitrilase from *Pseudomonas fluorescens* EBC191 also produced amide as a byproduct. The other highly enantioselective mandelonitrile hydrolases from *Alcaligenes* sp. ECU0401, *Alcaligenes faecalis* JM3, *Aspergillus niger* CBS 513.88, *Neurospora crassa* OR74A, *Pseudomonas putida* and *Alcaligenes faecalis* ATCC8750 exhibited a relatively low specific activity (Table 4). Thus, when the specific activity, enantioselectivity, and production of unwanted byproducts were taken into account, BCJ2315 demonstrated a great potential for the industrial production of optically pure (R)-(−)-mandelic acid.

### Conversion of mandelonitrile by the recombinant *E. coli* M15/BCJ2315

**Figure 4:**
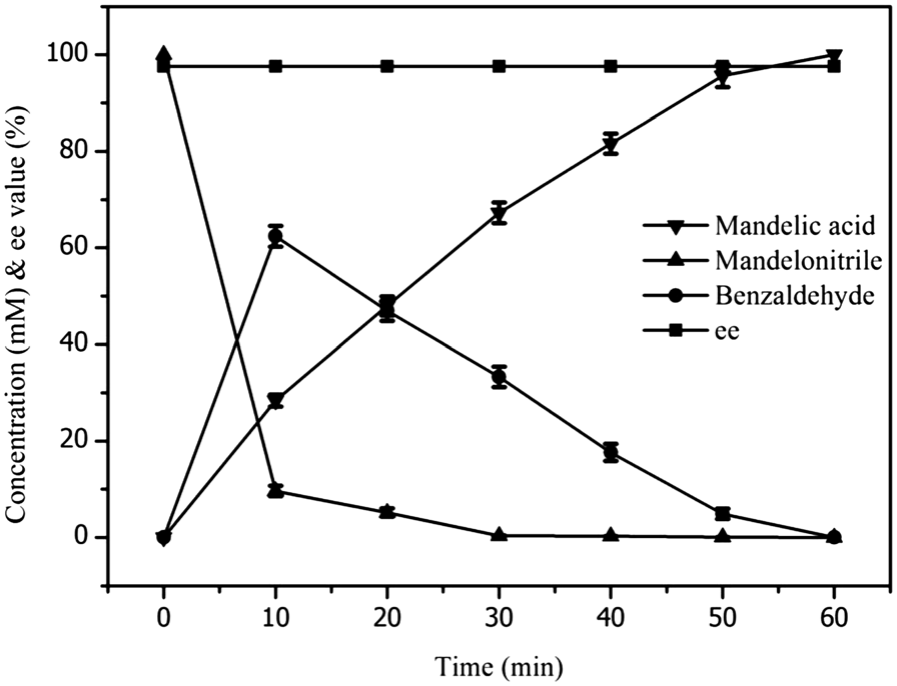
**Time course of the mandelonitrile hydrolysis mediated by the recombinant *E. coli *M15/BCJ2315.** The reaction mixture (10 ml) containing wet cells (100 mg) and mandelonitrile (100 mM) suspended in phosphate buffer (100 mM, pH 8.0) was incubated in a rotary shaker (30°C, 200 rpm). Samples were taken every 10 min and quenched by the addition of 10% (v/v) 2 M HCl.

Whole cell biocatalysis was performed using the recombinant *E. coli* M15/BCJ2315 as biocatalyst to estimate the potential of BCJ2315 for mandelonitrile hydrolysis further. An optimization of the reaction conditions for M15/BCJ2315 was performed by evaluating the effect of pH, temperature, cell concentration, and mandelonitrile concentration in a reaction system of 10 ml. The optimal reaction system consisted of wet cells (100 mg) and mandelonitrile (100 mM) in 10 ml of phosphate buffer (100 mM, pH 8.0) (data not shown). Although the optimal temperature was 45°C, we conducted the reaction at 30°C to consider the thermal deactivation of the enzyme. The reaction process is shown in Figure 4. Considering that mandelonitrile can be decomposed into benzaldehyde and hydrogen cyanide in aqueous solution at pH 7.0 and above, the benzaldehyde concentration was also plotted into the figure to help understand the mechanism of the mandelonitrile hydrolysis mediated by M15/BCJ2315. The mandelonitrile (100 mM) could be hydrolyzed completely by M15/BCJ2315 within 1 h. No mandelamide was detected during the reaction. The ee value for the (R)-(−)-mandelic acid was constant at 97.6% during the whole hydrolysis process. Finally, the (R)-(−)-mandelic acid was recovered with a total yield of 93.5%. The ee of the production was determined as 99.8% after the recrystallization in benzene through HPLC. The product was characterized as follows: [α]_D_^25^ = −152.7 (c 1.0, H_2_O) (literature: [α]_D_^25^ = −155 (c 1.0, H_2_O) [10]; ^1^H NMR (400 MHz, DMSO): δ12.58 (brs, 1H), 7.45–7.39 (m, 2H), 7.58 (d, *J* = 7.2 Hz, 2H), 7.32–7.26 (m, 1H), 5.84 (brs, 1H), 5.03 (s, 1H) ppm.

Among the reported nitrilase-mediated hydrolysis of mandelonitrile, only the nitrilase from *Alcaligenes* sp. ECU0401 could enantioselectively hydrolyze mandelonitrile in a high substrate concentration [36]. When 100 mM of mandelonitrile was used, the yield of the (R)-(−)-mandelic acid reached 100% with wet cells (100 mg/ml) in 2 h and the ee was as high as 99%. In the current study, we used less biocatalysts (10 mg/ml of wet cells) to realize the hydrolysis of the same mandelonitrile concentration (100 mM) with a high enantioselectivity (97.6%) in 1 h of reaction time. This result may be due to the high specific activity of BCJ2315 and the high soluble expression in *E. coli*. The biocatalyst preparation accounts for a great part of the total cost of an enzymatic process. Therefore, using M15/BCJ2315 for the production of (R)-(−)-mandelic acid is beneficial in two ways, i.e., the processing time is shorter and require less amount of biocatalyst (resting cells), thereby significantly reducing the production cost compared with other reported systems.

## Conclusions

A novel mandelonitrile hydrolase BCJ2315 was discovered from *Burkholderia cenocepacia* J2315 through PESSP. BCJ2315 took mandelonitrile as its optimal substrate and exhibited great advantages in the production of optically pure (R)-(−)-mandelic acid. These advantages include a high enantioselectivity and activity, strong substrate tolerance, and having no byproduct. These advantages make BCJ2315 more efficient in the hydrolysis of mandelonitrile. Thus, BCJ2315 has a great potential in the practical production of optically pure (R)-(−)-mandelic acid.

## Methods

### Materials

All the bacterial strains used for the mandelonitrile hydrolase analysis were obtained from the China General Microbiological Culture Collection Center, German collection of Microorganisms and Cell Cultures, and American Type Culture Collection (ATCC). *E. coli* BL21/pET28a(+) and M15/pQE30 were used for expressing the nitrilases. The nitrile substrates and carboxylic acids were purchased from Sigma–Aldrich (Milwaukee, USA).

### Database mining and sequence analysis

The database searches of the sequence data were performed using the BLASTP program (http://blast.ncbi.nlm.nih.gov/Blast.cgi?PAGE=Proteins&PROGRAM=blastp&BLAST_PROGRAMSblastp&PAGE_TYPE=BlastSearch&SHOW_DEFAULTS=on&LINK_LOC=blasthome). The nitrilase (bll6402, NP_773042) from *Bradyrhizobium japonicum* USDA110 [21] was chosen as the identifier to detect mandelonitrile-specific nitrilase (mandelonitrile hydrolase). Bll6042 was the most active toward mandelonitrile and took mandelonitrile as its optimal substrate. The identity of the amino acid sequence was used as the first criterion to screen the BLASTP result. Sequences with identities greater than 90% and less than 30% were removed to filter the enzymes with the same characteristics or with more distinct characteristics. No more than two sequences with the same identity were kept for analysis. The second criterion was the source of the sequence. Sequences from the same species were chosen once because these sequences usually share high identities and the same characteristics with one another. Two or more different sequences from the same strain were kept. The third criterion was the availability of the organisms that harbor nitrilase. Regardless of these three criteria, the sequences with experimentally defined nitrilase substrate specificity were chosen as a priority to refine the phylogenetic analysis. The sequences annotated as unnamed protein products were checked for the Glu-Lys-Cys catalytic triad [32,37] using the ScanProsite tool in the ExPASy proteomic server.

The alignment of the obtained sequences was conducted using ClustalW [38]. A bootstrap consensus tree was built by using a neighbor-joining method packaged in MEGA version 4.0 [39].

### Cloning and expression of nitrilase genes in *E. coli*

The nitrilase gene primers in the predicted mandelonitrile hydrolase subgroup used in this study are listed in Table 5. Recombinant DNA techniques were performed according to standard protocols [40]. All the recombinant expression plasmids were transformed into *E. coli* BL21 (DE3) or *E. coli* M15. The recombinant *E. coli* cells were cultivated in a Luria–Bertani medium containing antibiotics at 37°C. IPTG was added to a final concentration of 0.1 mM to induce the cultures when the OD_600_ reached 0.6 to 0.8. The *E. coli* BL21 (DE3) or *E. coli* M15 cultures were further incubated for 20 h at 20°C or 30°C, respectively. The induced cells were harvested through centrifugation (12,000 rpm, 10 min) at 4°C and stored at −20°C.

**Table 5:** Primers and locus tags of the nitrilase genes in the predicted mandelonitrile hydrolase subgroup used in this study

**Sequence Accession no.**	**Locus_tag**	**Source**	**Primer pair (sequence 5’→ 3’)**	**Restriction enzymes**
CP000699	Swit_0987	*Sphingomonas wittichii* RW1^a^	Forward: CATATGTCGGACGGTCCGTTCAAGGTTG	*Nde*I/*Hind*III
			Reverse: AAGCTTTTAGGAACCGGCCTTTTCGGCGA	
CU234118	BRADO4539	*Bradyrhizobium* sp. ORS 278^a^	Forward: GGGAATTCCATATGGGACTGGCACATCCG	*Nde*I/*Hind*III
			Reverse: GAAAAAAGCTTACCCGCCAGCCGCGACCT	
CP000494	BBta_4766	*Bradyrhizobium* sp. BTAi1^b^	Forward: GGATCCATGGGACTGGCACATCCGAAATAC	*Bam*HI/*Hind*III
			Reverse: AAGCTTCAGATGGATTGATCGGGCCGCGCG	
AM747720	BCAL2585	*Burkholderia cenocepacia* J2315^b^	Forward: GGATCCATGACCATCAATCACCC	*Bam*HI/*Hind*III
			Reverse: AAGCTTAAGCGGGTGTGACGC	
CP000555	Mpe_A0993	*Methylibium petroleiphilum* PM1^a^	Forward: CATATGCCGGTTTCGCACCCCAAG	*Nde*I/*Hind*III
			Reverse: AAAAAAGCTTAAGCCGTGCGGCGCGCGGTC	
BAD58116	nfa32690	*Nocardia farcinica* IFM 10152^a^	Forward: CATATGAGTCAGCGAGACAGTTTCCG	*Nde*I/*Hind*III
			Reverse: ATATAAGCTTCCGCACCGCGGGTTCGGCGT	
NZ_AAUW01000019	SIAM614_26356	*Stappia aggregata* IAM 12614^a^	Forward: CATATGAAAGCTATCAAGGTTGCCGCCGTTC	*Nde*I/*Bam*HI
			Reverse: GGATCCCTACTCCTCGACCTCAAAAGGCCGT	

^a^ The gene was cloned into a pET28a(+) expression vector using *E. coli* BL21 (DE3) as host strain.

^b^ The gene was cloned into a pQE30 expression vector using *E. coli* M15 as host strain.

### Protein purification

The obtained cell pellets were resuspended in 10 ml of ice-chilled lysis buffer (50 mM NaH_2_PO_4_, 300 mM NaCl, 10 mM imidazole, 1 mM dithiothreitol (DTT), pH 8.0). A cell disruption was performed by sonication on ice, and the lysate was centrifuged at 10,000 × g for 30 min to remove the cell debris. The resulting supernatant was passed through a 0.22-μm filter, and then applied to a Ni-NTA Superflow column (1 ml, Qiagen) previously equilibrated with the lysis buffer. The column was subsequently washed with 10 ml of wash buffer (50 mM NaH_2_PO_4_, 300 mM NaCl, 20 mM imidazole, 1 mM DTT, pH 8.0) to remove the impurity protein. The fusion protein was then eluted with the elution buffer (50 mM NaH_2_PO_4_, 300 mM NaCl, 250 mM imidazole, 1 mM DTT, pH 8.0). The eluted protein was desalted and concentrated through ultrafiltration using a 50-ml Amicon Ultra Centrifugal Filter Device with a molecular weight cutoff of 10 KDa (Millipore, USA). The purified enzyme was resuspended in a sodium phosphate buffer (pH 7.0) containing 1 mM of DTT and 20% glycerol, and then stored at −40°C. The crude extract and the pure enzyme were analyzed by SDS-PAGE. Protein concentration was determined using the Bradford method, with bovine serum albumin as the standard. All purification steps were carried out at 4°C.

### Enzyme assay

The standard reaction with the purified nitrilase was performed at 30°C in a reaction mixture (1 ml) containing 100 μmol sodium phosphate (pH 7.0), 20 μmol of nitrile substrate, and an appropriate amount of nitrilase. Aliquots (100 μl) were withdrawn at different time intervals, and 10 μl of 2 M HCl was added to quench the reaction. The production and optical purity of the mandelic acid were determined through an HPLC analysis. In some cases, the amount of ammonia formed in the reaction was measured by performing the Berthelot assay [41]. All the experiments were performed in triplicates. One unit of the enzyme activity was defined as the amount of enzyme that produced 1 μmol of mandelic acid or ammonia per min under the standard assay conditions.

### Determination of the molecular weight

The molecular weight of the purified BCJ2315 was determined by gel filtration chromatography on a Superdex 200 10/300 GL column (GE Healthcare). The calibration of the column was performed with the HMW-Gel Filtration Calibration Kit (GE Healthcare) containing thyreoglobulin (669 kDa), ferritiin (440 kDa), aldolase (158 kDa), and conalbumin (75 kDa). The void volume was determined using Blue Dextran (2,000 kDa).

### Effects of temperature and pH on the purified BCJ2315 activity

The reaction was performed at different temperatures or in buffers of different pH values for 10 min with 20 μg of purified BCJ2315 and 20 mM mandelonitrile in 1 ml of reaction mixture to determination the temperature and pH effects. The optimum temperature of BCJ2315 was determined by incubating the enzyme with mandelonitrile at different temperatures (18°C to 70°C). The optimum pH of BCJ2315 was determined by measuring the enzyme activity in buffers with different pH values (4.0 to 10.6) using mandelonitrile as the substrate. Sodium citrate-citric acid buffer (pH 4.0 to 6.4, 0.1 M), sodium phosphate buffer (pH 6.4 to 7.6, 0.1 M), Tris–HCl buffer (pH 7.0 to 9.0, 0.1 M), and Glycine-sodium hydroxide buffer (pH 8.5 to 10.6, 0.1 M) were used in this process.

### Measurement of the kinetic parameters

The kinetic parameters of BCJ2315 were determined over a wide range of mandelonitrile concentrations (0.1 mM to 15 mM) under standard assay conditions. The kinetic constants *V*_max_ and *K*_m_ were calculated from the Lineweaver– Burk plots using standard linear regression techniques.

### Substrate specificity

The specific activities of BCJ2315 toward different nitriles with structural diversity were measured under standard conditions. The reaction was incubated at 30°C for 5 min to 240 min. The conversion was determined by measuring the amount of ammonia produced in the reaction using the Berthelot assay, as described previously.

### Enantioselective mandelonitrile hydrolysis with the recombinant *E. coli* M15/BCJ2315

For the recombinant *E. coli* M15/BCJ2315, a standard reaction mixture (10 ml) containing wet cells (100 mg) and mandelonitrile (100 mM) suspended in phosphate buffer (100 mM, pH 8.0) was incubated in a rotary shaker (30°C, 200 rpm). Aliquots (100 μl) were withdrawn and quenched with 10 μl of 2 M HCl at different time intervals. The production and optical purity of the mandelic acid were determined by HPLC analysis. (R)-(−)-mandelic acid was recovered using an ion-exchange process, as described by Xue et al. [42]. The product was recrystallized using benzene as the solvent.

### Analytical methods

The decrease in mandelonitrile and the mandelic acid formation were analyzed by HPLC using a Zorbax SB-Aq column (4.6 mm × 250 mm, 5 μm) (Agilent Technologies, Ltd., USA) at a flow rate of 1 ml/min, with an eluting solvent system of phosphoric acid (0.1%, v/v) and methanol (20:80, v/v). A210 nm was measured.

The optical purity of the mandelic acid was determined by analyzing the enantiomers on a CHIRALCEL-OD-H column (4.6 mm × 250 mm, 5 μm) (Daicel Chemical Industries, Ltd., Japan) at a flow rate of 0.8 ml/min, with an eluting solvent system of hexane, isopropanol, and trifluoroacetic acid (95:5:0.1, v/v). A228 nm was measured.

## Competing interests

The authors declare that they have no competing interests.

## Authors’ contributions

DW and HW designed the study. HW carried out the bulk of the experiments. HS contributed to the expression and purification of the nitrilases. All authors have read and approved the final manuscript.

## Supplementary Material

Additional file 1: Table S1 The respective gene primers and mandelonitrile hydrolase activity of the nitrilases outside the predicted mandelonitrile hydrolase subgroup. Click here for file
