# Supplementary material for: Discovery and characterization of a highly efficient enantioselective mandelonitrile hydrolase from Burkholderia cenocepacia J2315 by phylogeny-based enzymatic substrate specificity prediction
Source: BMC Biotechnol. 2013 Feb 18;13:14. doi: 10.1186/1472-6750-13-14 (PMC3599355; doi:10.1186/1472-6750-13-14)
Supplement: Additional file 1: Table S1 — The respective gene primers and mandelonitrile hydrolase activity of the nitrilases outside the predicted mandelonitrile hydrolase subgroup. [file 1472-6750-13-14-S1.doc]

| Nitrilase | Sequence  Accession no. | Locus_tag | Source | Primer pair (sequence 5’→ 3’) | Restriction enzyme | Conversion of mandelonitrile(%)^a^ |
| --- | --- | --- | --- | --- | --- | --- |
| SWRW12 | NC_009511 | Swit_4178 | *Sphingomonas wittichii* RW1 | Forward: CATATGAACGAAGGCTTCCAGAAGGTAAGG | *Nde*I/*Hind*III | 1.7 |
|  |  |  |  | Reverse: AAGCTTTCAATCGTCGACGACGATCGGCAG |  |  |
| NF181 | XM_001261814 | NFIA_095380 | *Neosartorya fischeri* NRRL 181 | Forward: CATATGACCAAAGTCCGTGTGGGAG | *Nde*I/*Hind*III | N.D. |
|  |  |  |  | Reverse: AAGCTTACTCCATGTCAACAACGCACTTGG |  |  |
| NCNRRL1 | XM_001276026 | ACLA_076410 | *Aspergillus clavatus* NRRL 1 | Forward: CATATGGCGACACCTTCCCCTACGGT | *Nde*I/*Hind*III | N.D. |
|  |  |  |  | Reverse: GAAGCTTATTGAACAACCTTCTGCTTCC |  |  |
| BBRB50 | BX640440 | BB1116 | *Bordetella bronchiseptica* RB50 | Forward: CATATGACCACACATCGAATCGC | *Nde*I/*Hind*III | <1 |
|  |  |  |  | Reverse: AAGCTTCTAGGGTTTGAGCGTGGTGAC |  |  |
| AATC1 | NC_008711 | AAur_0337 | *Arthrobacter aurescens* TC1 | Forward: GGGAATTCCATATGACCAAAGTAGCAGTAG | *Nde*I/*Hind*III | 1 |
|  |  |  |  | Reverse: GAAAAAGCTTATACGGTCGCAGCGCCGTT |  |  |
| BP7601 | NZ_ABRX01000005 | BAT_1888 | *Bacillus pumilus* ATCC 7061 | Forward: CCCCCATATGGAGGTTTTTAATATGAC | *Nde*I/*Bam*HI | N.D. |
|  |  |  |  | Reverse: GGATCCTTACACTTTTTCTTCAAGC |  |  |
| RP009 | NC_005296 | RPA1563 | *Rhodopseudomonas palustris* CGA009^b^ | Forward: CCATGGCTAAGTTGAAAGTCGCGGCAG | *NcoI*/*Hind*III | N.D. |
|  |  |  |  | Reverse: AAGCTTTCAGGCTCCGGCGTCGCCTGCA |  |  |
| BGC4D1M | NZ_ABLD01000011 | BgramDRAFT_3842 | *Burkholderia graminis* C4D1M | Forward: CATATGAAAGTTGTCAAAGCCG | *Nde*I/*Hind*III | <1 |
|  |  |  |  | Reverse: AAGCTTTCAGCGCGAACCTGCAACAG |  |  |
| MPPM12 | NC_008825 | Mpe_A3478 | *Methylibium petroleiphilum* PM1 | Forward: ACATATGAGTCCCCCTCGCACC | *Nde*I/*Hind*III | N.D. |
|  |  |  |  | Reverse: AAGCTTAGCCAGCCGCAACCCGAAG |  |  |
| BX400 | NC_007951 | Bxe_A1408 | *Burkholderia xenovorans* LB400 | Forward: CATATGTCCGACAAATCGACCGGCAAG | *Nde*I/*Hind*III | N.D. |
|  |  |  |  | Reverse: AAGCTTAGTCATCAATTGCCGGCTCTCTG |  |  |
| RS101 | NZ_DS999213 | RGAI101_520 | *Roseobacter* sp. GAI101 | Forward: CATATGTCCCGTACCATCAAG | *Nde*I/*Hind*III | N.D. |
|  |  |  |  | Reverse: AAGCTTAATCCTCCAATGGGA |  |  |
| PPPF5 | NC_004129 | PFL_3461 | *Pseudomonas protegens* Pf-5 | Forward: CATATGATCAGCAAATGCGAAAAAACAGT | *Nde*I/*Hind*III | N.D. |
|  |  |  |  | Reverse: AAGCTTTCAGTCGTCGCGCAGCGTTAAAC |  |  |
| BAAMMD | NC_008390 | Bamb_0595 | *Burkholderia ambifaria* AMMD | Forward: CATATGTCCACCTCAGTCATCGC | *Nde*I/*Hind*III | N.D. |
|  |  |  |  | Reverse: AAGCTTAGCCTTCGAACACCACC |  |  |
| SP700345 | NC_009901 | Spea_2863 | *Shewanella pealeana* ATCC 700345 | Forward: GGATCCATGAAAAAAGTCGCAATTATTCAAG | *Bam*HI*/Not*I | 1.3 |
|  |  |  |  | Reverse: GCGGCCGCTTAGCTCTGATTCTTAAAGCTGA |  |  |
| BPSTM815 | NC_010623 | Bphy_5177 | *Burkholderia phymatum* STM815 | Forward: GGATCCATGTTGCATATGCAAGATCGGAC | *Bam*HI*/Hind*III | <1 |
|  |  |  |  | Reverse: AAAAGCTTTCATGCCGCTTGGGCGCGCGGAT |  |  |
| ZMZM4 | AE008692 | ZMO1207 | *Zymomonas mobilis* subsp. mobilis ZM4 | Forward: CATATGAGCTGTCATCGCGTAGCAGTTATC | *Nde*I/*Hind*III | N.D. |
|  |  |  |  | Reverse: AAGCTTATCTAATTGAGAGATCTCACTGTCG |  |  |

**Table S1.** **The respective gene primers and mandelonitrile hydrolase activity of nitrilases outside the mandelonitrile hydrolase subgroup**

^a^ The reaction was performed at 30°C for 12h in a reaction mixture (1 ml) containing 100 µmol sodium phosphate (pH 7.0), 20 µmol of mandelonitrile and 30 µg of purified nitrilase.

^b^ This nitrilase gene was cloned into pET42a(+) expression vector. The rest of nitrilase genes in this table were all cloned into pET28a(+) expression vector.
